# Gamified Exercise with Kinect: Can Kinect-Based Virtual Reality Training Improve Physical Performance and Quality of Life in Postmenopausal Women with Osteopenia? A Randomized Controlled Trial

**DOI:** 10.3390/s24113577

**Published:** 2024-06-01

**Authors:** Saima Riaz, Syed Shakil Ur Rehman, Danish Hassan, Sana Hafeez

**Affiliations:** 1 Riphah College of Rehabilitation and Allied Health Sciences, Riphah International University, Lahore 54000, Pakistan; shakil.urrehman@riphah.edu.pk (S.S.U.R.); danish.hassan@riphah.edu.pk (D.H.); 2 School of Health Sciences, University of Management and Technology, Lahore 54000, Pakistan; sana.hafeez@umt.edu.pk

**Keywords:** bone loss, postmenopausal, physical performance, osteopenia, quality of life, women

## Abstract

Background: Osteopenia, caused by estrogen deficiency in postmenopausal women (PMW), lowers Bone Mineral Density (BMD) and increases bone fragility. It affects about half of older women’s social and physical health. PMW experience pain and disability, impacting their health-related Quality of Life (QoL) and function. This study aimed to determine the effects of Kinect-based Virtual Reality Training (VRT) on physical performance and QoL in PMW with osteopenia. Methodology: The study was a prospective, two-arm, parallel-design, randomized controlled trial. Fifty-two participants were recruited in the trial, with 26 randomly assigned to each group. The experimental group received Kinect-based VRT thrice a week for 24 weeks, each lasting 45 min. Both groups were directed to participate in a 30-min walk outside every day. Physical performance was measured by the Time Up and Go Test (TUG), Functional Reach Test (FRT), Five Times Sit to Stand Test (FTSST), Modified Sit and Reach Test (MSRT), Dynamic Hand Grip Strength (DHGS), Non-Dynamic Hand Grip Strength (NDHGS), BORG Score and Dyspnea Index. Escala de Calidad de vida Osteoporosis (ECOS-16) questionnaire measured QoL. Both physical performance and QoL measures were assessed at baseline, after 12 weeks, and after 24 weeks. Data were analyzed on SPSS 25. Results: The mean age of the PMW participants was 58.00 ± 5.52 years. In within-group comparison, all outcome variables (TUG, FRT, FTSST, MSRT, DHGS, NDHGS, BORG Score, Dyspnea, and ECOS-16) showed significant improvements (*p* < 0.001) from baseline at both the 12th and 24th weeks and between baseline and the 24th week in the experimental group. In the control group, all outcome variables except FRT (12th week to 24th week) showed statistically significant improvements (*p* < 0.001) from baseline at both the 12th and 24th weeks and between baseline and the 24th week. In between-group comparison, the experimental group demonstrated more significant improvements in most outcome variables at all points than the control group (*p* < 0.001), indicating the positive additional effects of Kinect-based VRT. Conclusion: The study concludes that physical performance and QoL measures were improved in both the experimental and control groups. However, in the group comparison, these variables showed better results in the experimental group. Thus, Kinect-based VRT is an alternative and feasible intervention to improve physical performance and QoL in PMW with osteopenia. This novel approach may be widely applicable in upcoming studies, considering the increasing interest in virtual reality-based therapy for rehabilitation.

## 1. Introduction

Menopause is the cessation of a woman’s menstrual cycle, followed by 12 months of amenorrhea. The word “post-menopause” denotes the period following the cessation of the final menstrual cycle [1]. Menopause is a fundamental physiological phenomenon that manifests in women throughout the middle-age phase, signifying the cessation of a woman’s reproductive capacity. Natural menopause often manifests between 45 and 55. A correlation exists between premature menopause, elevated cardiovascular disease, and osteoporosis susceptibility. Conversely, delayed menopause increases the likelihood of developing breast and endometrial cancer [2].

Osteoporosis, also known as porous bone, is a medical condition defined by decreased bone density and the deterioration of bone structure. It makes weakened bones more prone to fractures, particularly in the hip and spine regions. Osteoporosis is characterized by a BMD that falls below the average level with a T-score of −2.5 standard deviations (SD) observed in young, healthy women [3]. Osteopenia is a precursor to osteoporosis and is distinguished by T-score values ranging from −1.0 to −2.5. At this juncture, interventions aimed at enhancing or preserving BMD are warranted [4].

The prevalence of osteoporosis in Asian women is higher than in Western countries, with estimates ranging from 25% to 38% in Asian populations, as opposed to 9% to 16% in Western populations [5]. Osteopenia or osteoporosis frequently impacts women in the postmenopausal stage, elevating their susceptibility to fractures [6]. PMW demonstrate a decline in estradiol synthesis and an elevation in follicle-stimulating hormone levels, resulting in accelerated bone remodeling [7]. In the early stages of menopause, women with lower bone mass have less handgrip muscle strength. Furthermore, during the later stages of menopause, these women experience a more pronounced reduction in BMD and functional performance [8]. Reduced back muscle strength was associated with higher bone loss in PMW who experienced early menopause [9]. The aging process and entering post-menopause can lead to accelerated bone loss and decreased muscle fiber quantity and size. Consequently, this increases the vulnerability to osteoporotic fractures [10] and the development of kyphosis, which can have enduring detrimental effects such as diminished back and lower limb functionality [11].

Bone mass is subject to adequate physical activity (PA), hormonal regulation, nutritional intake, and genetic predisposition. Early detection and prevention of further bone loss are imperative in diagnosing low bone mass. The application of appropriate mechanical stress has the potential to induce osteogenic activity [12].

Musculoskeletal health is significantly affected by sedentary behavior or insufficient levels of PA [13]. Similarly, it has been observed that the musculoskeletal system operates at its best when subjected to moderate physical activity and exercise [14,15,16]. PA is the engagement in incidental daily activities, such as walking for transportation, work-related tasks, household duties, and exercise. This sort of activity is conducted with a deliberate intention to achieve a specific goal or target [17]. Physical inactivity causes various physical and mental health problems in PMW, and these are linked with menopause. On the other hand, physical activities are considered vital for the physical and mental health of PMW [18].

Regularly promoting physical exercise among women after reaching middle age might effectively alleviate postmenopausal symptoms [18,19]. In addition, it is emphasized that implementing physical therapy can improve patients’ physical health and, as a result, their overall quality of life [19,20].

Exergames can be a viable alternative or supplementary approach to traditional training methods. The term “exergame” pertains to the engagement in dynamic activities designed to achieve objectives as directed by video games. Adopting this approach emphasizes the task’s aim, enhancing the external focus. As a result, the development of motor skills will progress rapidly towards the upper echelons of the rehabilitation pyramid [21].

The utilization of technology in rehabilitation is experiencing significant and swift growth. Hence, there is a current effort to create interactive systems involving patients in diverse therapeutic interventions. In light of recent technical advancements, Microsoft’s Kinect (Xbox 360 S CONSOLE, Microsoft Corporation M-1439, Redmond, WA, USA), has emerged as a cutting-edge user interface technology that effectively supports and enhances numerous clinical applications [22]. The Xbox 360 Kinect, an innovative video game technology that eliminates the need for traditional controls, has emerged as a novel inclusion in rehabilitation programs [23].

PMW with osteopenia have diminished physical performance, characterized by compromised balance, gait, and muscle strength, which results in functional limitations and increased vulnerability to falls and fractures, substantially impacting their QoL and their physical, emotional, and social well-being. Assessing the impact of osteoporosis and osteopenia management is crucial by considering health-related QoL. Despite numerous research studies examining the effects of exercise interventions on the QoL in PMW with osteoporosis or osteopenia, there is still a debate on whether exercise influences the health-related QoL in PMW. Due to the scarcity of research, inadequate sample size, and absence of high-quality studies, drawing definitive conclusions regarding the impact of exercise on QoL is challenging. Hence, additional elucidation regarding the importance of exercise training in PMW with osteopenia is necessary.

Conventional exercise interventions, while advantageous, may face challenges with adherence due to safety concerns, pain, and inadequate motivation. The utilization of Kinect-based VRT has become known as a promising alternative, providing captivating experiences that have the potential to overcome such challenges. VRT environments can gamify the exercise, enhancing its appeal and motivation and enabling training in the controlled virtual environment, thereby reducing the risk of falls and eliminating discomfort associated with specific exercises. This allows for immediate feedback and continuous progress monitoring, increasing engagement and motivation.

Our hypothesis posits that Kinect-based VRT utilization substantially impacts the physical performance and QoL of PMW diagnosed with osteopenia. Hence, this study aimed to determine the effects of Kinect-based VRT on physical performance and QoL in PMW with osteopenia.

## 2. Materials and Methods

### 2.1. Study Design and Study Settings

**Figure 1:**
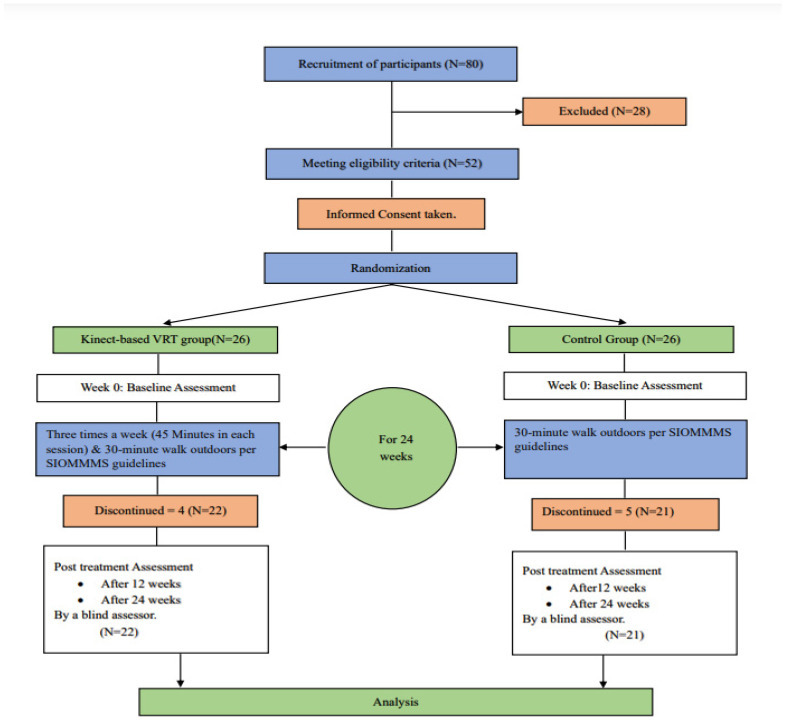
Consolidated Standards of Reporting Trials (CONSORT) Diagram.

The present study was a prospective, randomized, double-blinded controlled experiment with parallel groups, conducted between 2021 and 2023 at Riphah Rehabilitation Center, Riphah International University, Lahore, Pakistan, as well as at Genesis Healthcare Consultants, also located in Lahore, Pakistan. The clinical trial protocol received ethical approval from the Research and Ethics Committee at Riphah College of Rehabilitation and Allied Health Sciences, Riphah International University, Lahore, Pakistan (REC/RCR&AHS/21/1102). The study was conducted under the principles outlined in the Helsinki Declaration. The clinical study with the identifier NCT04862910 was officially registered in the Clinical Trials.gov Protocols Registration and Results System on 28 April 2021. Each patient gave written consent to participate in the trial. Figure 1 provides a summary of the study design.

### 2.2. Sample Size

The recruitment of study participants was done using non-probability convenience sampling. The sample size was selected based on data from pilot research. The pilot trial included twenty subjects, with the experimental group having a mean lumbar spine BMD of 0.03 ± 0.02 and the control group having a mean lumbar spine BMD of 0.007 ± 0.032. The preliminary investigation resulted in an effect size of 0.9. The sample size of 42 was calculated based on an effect size of 0.9, 80% statistical power, a 95% confidence interval (CI), and a 5% margin of error. After factoring in a 20% attrition rate, a sample size of 52 was established, with 26 participants assigned to each group.

### 2.3. Study Participants

A sample of 52 PMW was carefully selected and recruited using various promotional techniques, including posters, brochures, social media campaigns, and outpatient physical therapy clinics. The purpose was to assess and figure out their bone health condition. The Simple Calculated Osteoporosis Risk Estimation (SCORE) score of greater than 6 indicated a recommendation for a Dual-Energy X-ray Absorptiometry (DEXA) scan [24]. The inclusion criteria for participants required a serum calcium level within the normal range of 8.6–10.3 mg/dL, as well as a minimum serum 25-OH vitamin D value of 30 ng/mL [25], must be aged between 48 and 70 years old, must have been diagnosed with osteopenia using dual-energy X-ray absorptiometry, with a T score between −1 and −2.5 in either the lumbar spine or femur, diagnosis must have been recommended by the study investigators or confirmed by a doctor within the past 12 months. Additionally, participants must have a current body mass index (BMI) of 30 kg/m^2^, should have a normal balance and no risk of falling, as assessed by the Tandem Stance Test (TST), to rule out fear of falling, participants should have a Fall Efficacy scale (FES-I) value between 18–28 [26]; and osteopenic PMW with or without medication. Patients who had severe sensory and/or communication impairments, cognitive impairments, unstable angina, lung conditions requiring oxygen therapy, any serious medical condition, neurological problems, a history of virtual game therapy within the past six months, virtual game phobia, simulator or motion sickness, symptomatic orthostatic hypotension (diastolic blood pressure > 95 mmHg, systolic blood pressure > 160 mmHg), secondary osteoporosis, arthrosis, known osteoporotic fractures, neoplastic illness, or epilepsy were not included in the study. An unbiased assessor examined all enrolled participants in the initial evaluation to ascertain their eligibility for inclusion in the study. The subjects underwent three assessments throughout the study: in the beginning, after 12 weeks, and again after 24 weeks following the intervention. Before conducting the baseline examination, participants were instructed to acquaint themselves with the study method and obtained informed consent by signing a consent form. The participants were explicitly notified of their right to withdraw from the research without encountering any adverse repercussions and were actively encouraged to seek clarity by posing inquiries.

### 2.4. Group Allocation by Randomization

Participants who provided informed consent and expressed their willingness to participate in the research were allocated randomly to one of two groups using a computer-generated randomization table (Figure 1). The sequentially numbered, opaque, sealed envelopes (SNOSE) method was used to disguise the allocation. The envelopes were designed by an impartial researcher who had no clinical involvement. All personnel, except for the therapist providing treatment, were unaware of the treatment, including the assessor and participants. The participants were allocated to either the Experimental or Control Group.

#### 2.4.1. Control Group

The control group was directed for a daily 30-min outdoor walk, adhering to the guidelines set forth by the Societa’ Italiana dell’Osteoporosi, del Metabolismo Minerale e delle Malattie dello Scheletro (SIOMMMS), which is the Italian Society for Osteoporosis, Mineral Metabolism, and Bone Diseases [27,28]. However, they did not receive structured and monitored exercises throughout the same period and were advised to continue their regular lifestyle [29,30]. Regular communication was maintained by adjusting the participants’ monthly visits and phone calls [31].

#### 2.4.2. Experimental Group

The experimental group participated in Kinect-based VRT for 45 min per day, three times per week, on alternating days, over 24 weeks. In addition, they were directed to partake in a daily 30-min outdoor walk, adhering to the SIOMMMS recommendations.

Xbox Kinect (Xbox 360 S CONSOLE, Microsoft Corporation M-1439, Redmond, WA, USA), a gaming device, was utilized for the Kinect-based VRT. An LED television display was positioned in a serene environment to facilitate active video game training using Xbox Kinect, while the gaming device’s infrared camera sensor was placed in front of the monitor. The participants were approximately 1.5–2 m from the monitor [32].

Games for the Kinect were chosen to be played three times per week. A pilot study was done to determine the effect size of Virtual Reality Therapy (VRT) using Kinect technology on PMW diagnosed with osteopenia. The Xbox Kinect 360 game program selection considered the participant’s upper and lower body strength, balance, and high-impact training. The chosen programs, which included games from Your Shape Fitness Evolved, Kinect Sports, and Kinect Adventures (Table 1), gradually engaged the muscles of the lower extremities, upper limbs, and torso as the game’s intensity increased [32]. Each game was played for the designated duration. The games were selected based on their difficulty level, and as the participants’ performance improved, the difficulty level gradually increased. During gameplay, the participants observed an animated character on the LED screen that mimicked their movements. To achieve a high score in the game, the players had to synchronize their actions by closely monitoring the character’s movements [33]. The participants were informed about the score from the previous segment and encouraged to update it in the next section of the game. Before the commencement of game training and if assistance was required, the therapist allowed the participants to select a game while closely monitoring them to ensure their well-being. The clinical implementation of the Kinect-based VRT technique occurred in three sequential steps [34]:

**STEP I:** Warm Up
Track and Field (Kinect Sports)----------------5 min.
Step Touch & Tap back (Your shape Fitness evolved)-----5 min.
**STEP II:** The participant engaged in the games under the therapist’s guidance three days per week over 24 weeks. Participants were involved in different games alternating weekly days, providing enjoyment and motivation. This approach prevented boredom that could arise from repeating the same games (Table 1).
**STEP III:** Cool Down
Track and Field (Kinect Sports)----------------5 min.

**Table 1:** Weekly program of Kinect-Based VRT Games.

Days of a Week	Kinect Games	Aim	Specific Games	Time
Day 1	Your Shape Fitness Evolved	suitable for training older adults’ static and dynamic balance and lower-body endurance and strength [35]	Loop a Hoop Wall Breaker	15 min 15 min
Day 2	Kinect Sports	High-impact training with weight transfer for the lower body, static balance, lateral trunk stability, reaction speed, progressive reaching, and strength training of the upper body [36]	Bowling Boxing Soccer Beach volleyball Foot Ball Skiing	5 min 5 min 5 min 5 min 5 min 5 min
Day 3	Kinect Adventures	Trunk control, coordination, reaction speed, weight transfer (Impact training), balance and posture-holding [36]	20,000 leaks (all levels) River Rush Reflex Ridge	10 min 10 min 10 min

### 2.5. Outcome Measures

#### 2.5.1. Anthropometric Measurements

The body height was measured using a stadiometer (TKK 11253, Takei Scientific Ins Co., Tokyo, Japan) with an accuracy of 0.1 cm. The body weight was measured using a Seca 700 balance beam scale manufactured by Seca Co. in Hamburg, Germany. The scale provided measurements accurate to the nearest 0.1 kg. The researchers calculated the body mass index (BMI) by dividing the body weight (in kilograms) by the square of the body height (in square meters).

#### 2.5.2. Time Up and Go Test (TUG)

The TUG test was conducted twice, recording the duration in seconds. The participant stood up from a back-supported chair, walked 3 m, reversed their direction, returned to the chair, and resumed their seated position. The outcome of the best trial was recorded. A stopwatch was utilized to measure the duration required to finish the test [1].

#### 2.5.3. Functional Reach Test (FRT)

The FRT is a method used to assess physical performance outcomes by measuring dynamic balance through a simple task [37]. The participants positioned themselves barefoot, with their feet spaced apart at shoulder width, aligning their toes with a defined baseline on the floor. Subsequently, participants were instructed to elevate their arms until parallel to the floor. The position of their fingers at this point was documented as the initial position. Subsequently, participants were directed to extend their arms as far as possible in front of them while maintaining their position without shifting or raising their feet or compromising their stability. The resulting measurement, in centimeters (cm), was then documented. Two experiments were conducted, and the longest distance was chosen for statistical analysis.

#### 2.5.4. Five Times Sit to Stand Test (FTSST)

The Five Times Sit to Stand Test (FTSST) is a standardized and clinically formulated performance-based test created by Csuka and McCarty. The primary objective of utilizing the test is to evaluate the strength of the lower limbs and the balance and postural control in an aged population with high reliability (intraclass correlation coefficient (ICC) range: 0.914–0.933) and excellent test-retest reliability (ICC range: 0.988–0.995) [38,39]. It involved standing up from a chair five times as fast as possible without pushing off. The participant was given instructions regarding the performance before the test, standardized verbal instructions during the performance, and two test practice opportunities. The participants sat comfortably with their backs supported by the chair and arms folded in front of their chest during the exam. The performance chair was 45 cm tall. Participants were instructed to rise from the chair by extending their knees and avoiding bouncing movements. A stopwatch measured the duration of the performance, from the moment it started until it ended, while the performer was seated.

#### 2.5.5. Modified Sit and Reach Test (MSRT)

In addition, the Modified sit-and-reach test assessed lower back and hamstring flexibility. To eliminate shoulder girdle mobility and arm-leg differences administratively, Hopkins and Hoegers devised the Modified Sit and Reach Test (MSRT) [40]. Participants sat with their head, back, and hips against a wall (90° angles) and feet against a sit-and-reach box at hip distance. The participant was instructed to lay the right hand over the left and carefully reach ahead to the box’s measuring scale’s farthest extent. During early reach, only scapular abduction was done with the head and back against the wall. The sliding measurement scale was then pushed along the box top until the zero point was reachable by fingers. Due to limb length disparities, this technique determined the Fingers to Box Distance (FBD) as the relative zero point for each participant. Participants then slid forward to complete the reach test, which was scored by distance.

#### 2.5.6. Hand Grip Strength (HGS)

Hand Grip Strength (HGS) is a quantitative assessment of muscular strength, explicitly referring to the maximum force or tension produced by the muscles in one’s forearm. It is a screening tool for assessing the upper body and overall strength. The HGS of the dominant and non-dominant hand was evaluated by conducting three consecutive repetitions utilizing a Jamar hand dynamometer (Handexer Digital Grip Strength Tester 265 lb/120 kg/Digital Hand Dynamometer). The elbow was flexed at a 90° angle and maintained in a position where it did not make touch with any other part of the body. The interval between consecutive measurements was 30 s. The study utilized the average value of the two best performances [41].

#### 2.5.7. BORG Score

The Borg Score is a metric used to quantify an individual’s exertion level, dyspnea, and fatigue when performing a physical activity. The scale enables individuals to subjectively assess their level of exertion during exercise or exercise testing, as stated by the American College of Sports Medicine (2010). The Rate of Perceived Exertion (RPE)scale used in this study is the BORG-CR10, a category-ratio scale from 0 to 10, invented by Borg. The RPE scale quantifies the level of exertion during physical activity. The numbers correspond to the phrases employed to evaluate an activity’s difficulty or intensity level [42].

#### 2.5.8. Dyspnea Index

The Dyspnea Index was used as a clinical assessment to determine degrees of physical effort. The Baseline Dyspnea Index (BDI) quantifies the intensity of dyspnea at the initial stage of a clinical trial. The Transition Dyspnea Index (TDI) quantifies the alterations in dyspnea experienced during the transition phase compared to subsequent visits [43].

#### 2.5.9. ECOS-16

Escala de Calidad de vida Osteoporosis (ECOS-16) was originally developed in the Spanish language to evaluate the QoL in PMW with osteoporosis or osteopenia based on the osteoporosis-specific QoL instruments; the QoL Questionnaire of the European Foundation for Osteoporosis (QUALEFFO-41) and the Osteoporosis QoL Questionnaire (OQLQ) [44]. ECOS-16 contains 12 items from QUALEFFO and four items from OQLQ. The 16 items in ECOS-16 are divided into physical functioning, pain, fear of illness, and psychosocial function. Five response options are offered per item, with scores varying between 1 and 5 points, where 5 represents the worst QoL score.

### 2.6. Statistical Analysis

The statistical analysis was conducted using the SPSS statistical software (Version 25; IBM Inc., Chicago, IL, USA). The descriptive data was given as mean ± standard deviation, and intergroup differences of baseline characteristics were calculated using an Independent T-Test. The Shapiro-Wilk W-test was used to assess the normality of the sampling distribution for inferential analysis. The Repeated Measures Analysis of Variance (ANOVA) was employed to compare the mean changes in scores between and within groups to assess the intervention’s effects. The significance level was established at *p* ≤ 0.050. Mixed effects analysis of variance (ANOVA) was conducted to analyze the impact of Group (experimental and Control), Time (Baseline, 12 weeks and 24 weeks), and their interaction on each dependent variable. The effect size was computed using partial eta squared (η^2^p) for variance analysis. An η^2^ number ranging from 0.1 to 0.24 signifies a moderate effect level, while a value between 0.25 and 0.36 suggests a medium effect level. A value of 0.37 or higher indicates a large effect level.

## 3. Results

The study adhered to the CONSORT guidelines for reporting the trial. Initially, 80 PMW were assessed for eligibility, and subsequently, 52 PMW were randomly assigned, with 26 in each group. However, due to dropouts, the final evaluation included 22 participants from the experimental group and 21 from the control group. The data presented in Table 2 compare the baseline characteristics of the experimental group (n = 22) and the control group (n = 21). The ages of the participants ranged from 48 to 68 years. There were no statistically significant differences in the baseline characteristics between the two groups (*p* > 0.50), indicating that both groups were well-matched at the baseline.

Table 3 presents the frequency of both groups’ demographic characteristics, health issues, and lifestyle habits. Both groups exhibited nearly identical features, except for contraceptive usage, which was more prevalent in the control group. The experimental group had no preexisting fractures. The groups were probably highly compatible.

Table 4 shows intra-group and inter-group comparisons of outcome variables at baseline, 12th week, and 24th week. The intra-group comparison reveals a statistically significant change in all outcome variables using the within-group Repeated Measure ANOVA. In the experimental group, significant change in TUG (F_(2, 42)_ = 385.52, *p* < 0.001, partial Ƞ^2^ = 0.948), FRT (F_(2, 42)_ = 617.84, *p* < 0.001, partial Ƞ^2^ = 0.967), FTSST (F_(2, 42)_ = 329.29, *p* < 0.001, partial Ƞ^2^ = 0.940), MSRT (F_(2, 42)_ = 404.75, *p* < 0.001, partial Ƞ^2^ = 0.951), DHGS (F_(2, 42)_ = 382.77, *p* < 0.001, partial Ƞ^2^ = 0.948), NDHGS (F_(2, 42)_ = 253.18, *p* < 0.001, partial Ƞ^2^ = 0.923), BORG Score (F_(2, 42)_ = 395.67, *p* < 0.001, partial Ƞ^2^ = 0.950), Dyspnea Index (F_(2, 42)_ = 387.69, *p* < 0.001, partial Ƞ^2^ = 0.949) and ECOS-16 (F(_2, 42)_ = 819.21, *p* < 0.001, partial Ƞ^2^ = 0.975) was seen. While, in the control group, the change in the TUG (F_(2, 40)_ = 24.63, *p* < 0.001, partial Ƞ^2^ = 0.552), FRT (F_(2, 40)_ = 7.19, *p* = 0.002, partial Ƞ^2^ = 0.264), FTSST (F_(2, 40)_ = 36.61, *p* < 0.001, partial Ƞ^2^ = 0.605), MSRT (F_(2, 40)_ = 20.85, *p* < 0.001, partial Ƞ^2^ = 0.510), DHGS (F_(2, 40)_ = 16.51, *p* < 0.001, partial Ƞ^2^ = 0.452), NDHGS (F_(2, 40)_ = 20.57, *p* < 0.001, partial Ƞ^2^ = 0.507), BORG Score (F_(2, 40)_ = 235.11, *p* < 0.001, partial Ƞ^2^ = 0.922), Dyspnea Index (F_(2, 40)_ = 363.37, *p* < 0.001, partial Ƞ^2^ = 0.948) and ECOS-16 (F_(2, 40)_ = 157.90, *p* < 0.001, partial Ƞ^2^ = 0.890), was seen. After a 24-week intervention, both the experimental and control groups exhibited statistically significant improvements in all assessed variables, including TUG, FRT, FTSST, MSRT, DHGS, NDHGS, BORG Score, Dyspnea Index, and ECOS-16. There was a significant difference in each pair of times, except for the 12th to 24th week, for FRT in the control group. Nevertheless, the control group’s FRT exhibited a substantial average disparity from the initial measurement to the 24th week. The experimental group showed greater improvements in within-group analysis than the control group in most variables, indicating a potential advantage of Kinect-based VRT for enhancing physical performance and quality of life in PMW with osteopenia.

The inter-group comparison reveals the mean differences between the experimental and control groups using between-group analysis by ANOVA. The results showed a statistically significant difference between the two groups (*p* < 0.001), with a 95% confidence interval. The experimental group exhibited superior outcomes compared to the control group.

Table 5 presents the Mixed-effects analysis as Mean Squares (MS), F-values, Partial Eta-squared (Ƞ^2^), and *p*-values for each effect. The F-values and *p*-values (<0.001) validate the statistical significance of the impact, while the partial eta-square estimates the proportion of variance in the dependent variable accounted for by each effect. Except for the Borg Score and Dyspnea, most dependent variables significantly affect both Group and Time (*p* < 0.001 for F statistic). This implies that the dependent variables vary among groups and undergo changes over time. Furthermore, the presence of a significant interaction (Group*Time) for most variables suggests that the impact of time may vary across various groups. The statistical analysis indicates that there is a marginally significant interaction impact between the variables “Borg Score” and “Dyspnea” in the “Group*Time” interaction (*p*-value between 0.01 and 0.05).

**Figure 2:**
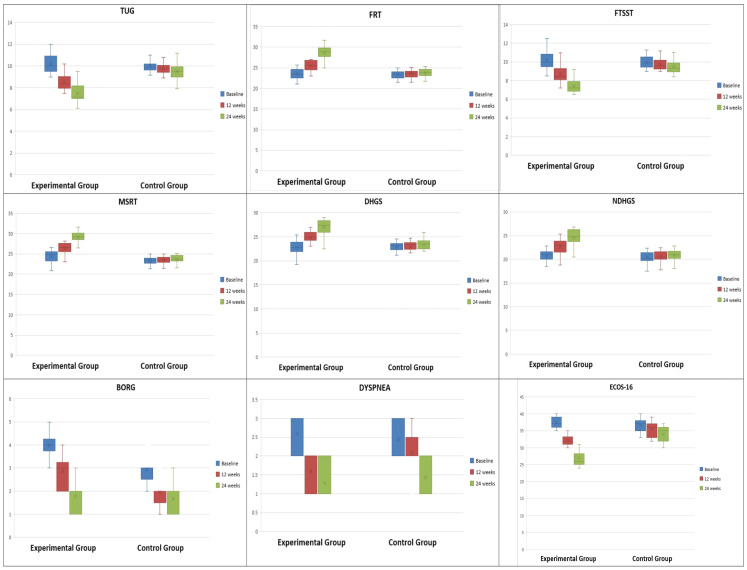
Estimated marginal means of TUG, FRT, FTSST, MSRT, DHGS, NDHGS, BORG Score, and Dyspnea across both groups at baseline, 12 weeks, and 24 weeks.

Figure 2 visually represents a group-wise comparison of estimated marginal means of all outcome variables at baseline, 12th week, and 24th week. The experimental group shows significantly better improvements compared to the control group in most aspects, such as faster TUG and FTSST times, higher reach distance in FRT, stronger DHGS and NDHGS, lower BORG scores, and reduced instances of dyspnea compared to the control group.

## 4. Discussion

The study’s objective was to assess the impact of Kinect-based VRT over 24 weeks on the physical performance and QoL in PMW with osteopenia. Conversely, the control and experimental group followed the SIOMMMS guidelines provided by the Italian Society for Osteoporosis, Mineral Metabolism, and Bone Diseases, which involved a daily 30-min outdoor walk [28,29]. Nevertheless, the control group was not provided with organized and supervised exercises and were instructed to maintain their usual daily routine [30,31]. Consistent communication was kept by modifying the frequency of the participants’ monthly visits and phone calls. Both groups demonstrated improvement; however, the experimental group had superior outcomes in terms of improvement.

Bieryla conducted a preliminary study where ten seniors with an average age of 70 engaged in Xbox Kinect motion-based training for three weeks. The study found that this exercise regimen modified the Berg and Fullerton advanced balance scales [45]. Babadi and Daneshmandi compared the effects of 9 weeks of VRT combined with traditional balance training in 36 elderly individuals. The study utilized various assessments, including the functional reach test, single leg stance with open/closed eyes, Fullerton advanced balancing test, and Timed Up and Go (TUG) test. The findings obtained from both types of interventions were shown to be similar when compared to the control group [46]. In the current study, 24 weeks of Kinect-based VRT was applied in 43 PMW with osteopenia aged 48–70 years, resulting in improved physical performance (measured by TUG, FRT, FTSST, MSRT, DHGS, NDHGS, BORG score and Dyspnea Index) and improved QoL (measured by ECOS-16 questionnaire).

Nambi et al. [47] studied the effects of Kinect-based VRT in PMW with osteoporosis. Participants played a Kinect-enabled Xbox 360 shooting game. It took 45 min per day and four days per week to complete the 12-week training program. In PMW with osteoporosis, VRT enhanced BMD, bone mineral content, and QoL. This training method also reduced osteoporotic fractures [47]. Our study used Kinect-based VRT to assess its impact on physical performance and QoL in osteopenia-afflicted PMW. In the VRT protocol, the warm-up, active phase, and cool-down phases were implemented sequentially. All stages were performed with evidence-based X-Box 360 Kinect games. The training was 45 min per day, thrice a week for 24 weeks, and the current study only included osteopenic PMW.

Shaw and Snow [47] found that weight-bearing resistance exercises for nine months significantly improved muscle strength and lateral stability in PMW between 50 and 75. In their study, Lord et al. [48] discovered that a 12-month training regimen that targeted the improvement of quadriceps muscular strength, balance, and coordination resulted in greater strength in the quadriceps muscles and reduced postural instability in older women. The present study utilized Kinect-based VRT that improved the TUG test, which assesses balance and fear of falling; the FRT, which evaluated dynamic balance through a straightforward task; and the FTSST, which measured lower limb strength, balance, and postural control in PMW with osteopenia.

The impact of aerobic exercise on balance and fall prevention is being extensively studied in PMW. Shigematsu et al. [49] found that engaging in a 3-month aerobic dance exercise program enhanced balance. Additionally, they demonstrated that a relatively brief submaximal aerobic exercise program (4 weeks, twice a week) significantly improved static and dynamic balances and mobility in PMW. We corroborated these reports, but with an extended duration of 24 weeks of training using Kinect-based VR technology, conducted thrice a week. This training resulted in significant improvements in various physical performance measures, such as static and dynamic balances, fear of falling, lower limb muscle strength, flexibility of hamstring and back muscles, hand grip strength, level of exertion and fatigue, and overall QoL in PMW with osteopenia.

The controlled multi-factorial exercise training program incorporates functional and static exercises. Young adults and athletes can engage in aerobics, running, skipping, dynamic strength training, isometric co-contraction exercises, and dancing to enhance their physical fitness. Engaging in high-impact sports such as jumping, running, squash, and tennis positively influences BMD, improving physical function and QoL. This effect contrasts with low-impact sports activities like swimming [12]. Population and intervention studies indicate that high-impact activities are more effective in raising BMD and improving QoL than low-impact activities. However, low-impact activities can still be beneficial in preventing further loss [13]. The core program exercises, as identified by Robinson RJ et al., consist of low-impact activities such as box steps, heel presses, step-step-kick-step-step-back, abdominal exercises, upper body twists, cross steps, knee lift triangles, leg presses, rowing exercises, bent side leg raise, bent leg raise, front leg tucks, and side leg presses [14]. Engaging in moderate aerobic activity with low impact can help prevent bone loss, while intense aerobic exercise with high impact can increase BMD and physical functioning [15]. The current study’s findings align with the research above, which suggests that Kinect-based VRT encompasses a blend of weight-bearing and high-impact, moderate-impact, and low-impact exercises. The effects of these exercises on physical performance and QoL are comparable to other weight-bearing and high-impact exercises, such as running, jumping, hiking, and skiing, as well as moderate-impact and low-impact exercises.

As per the SIOMMMS guidelines, the experimental and control groups were advised to engage in a 30-min outdoor walk as a therapeutic method. The control group likewise demonstrated enhancements in physical performance and QoL. The results of our investigation were supported by Nelson et al. [50], who asserted that all prescribed exercise regimens, including aerobic exercise, resistance training, and walking, have advantageous effects in decelerating the decline of BMD over a duration of one year or more and improving QoL. Fast walking is the most recommended approach for preventing and treating osteoporosis and enhancing the QoL in PMW. It closely resembles daily activities and is likely to be followed more consistently. In this study, we integrated a 30-min outdoor walk with Kinect-based virtual reality training (VRT) in the experimental group. This combination resulted in more significant improvements than the control group, who just engaged in a 30-min outdoor walk.

Kastelan et al. examined the impact of 24 weeks of aerobic exercises on BMD, physical fitness, and QoL in PMW diagnosed with osteoporosis. The data suggest that aerobic training can reduce fractures and decrease the likelihood of falling, which is attributed to enhanced BMD. Significant differences were seen between active participants and controls regarding pain, physical activity, social life, and subjective health, as indicated by statistical analysis [51]. Similarly, the present study found that a 24-week Kinect-based VRT had notable impacts on the physical performance and QoL of PMW with osteopenia.

A study on using Kinect-based games for patient rehabilitation emphasized the significance of integrating these games into rehabilitation programs owing to their ability to observe movement and control activity levels. Games employing Kinect technology proved highly efficacious in treating chronic diseases [52]. Zoccolillo et al. discovered that video-based gaming activity training with an Xbox 360 Kinect System significantly enhanced upper limb abilities compared to conventional therapy in children diagnosed with cerebral palsy [53]. Sevick et al. have confirmed that kinetic games substantially improve upper limb functions and promote motivation in children with cerebral palsy [54]. Consistent with previous research, our study found that participants using Kinect-based VRT improved their upper limb strength and function, as measured by hand grip strength. It is important to note that our research specifically focused on PMW diagnosed with osteopenia.

Although our study findings are promising, it is essential to acknowledge certain limitations. Participants needed to visit the setting three times a week, which resulted in a few cases dropping out. Establishing communication with the participants in the control group proved to be challenging. Several confounding variables, including medication usage, dietary factors, and comorbid medical conditions, could have an impact on the physical performance and QoL in PMW with Osteopenia. The intervention’s feasibility and effectiveness may have been compromised by technological limitations and obstacles associated with the Kinect-based VRT. These limitations include Tracking Accuracy and Fidelity, where the Kinect sensors may have difficulty capturing intricate movements, the system’s Limited Range of Motion may restrict the types of movements that the Kinect sensors can accurately capture, and the system’s performance can be influenced by Environmental Dependence, such as lighting conditions, background clutter, and the presence of multiple users in the same space which can impede the consistency and reliability of VRT.

It is suggested that Kinect-based VRT be utilized as an innovative treatment strategy to improve physical performance measures and QoL in PMW with osteopenia. Further studies, specifically with a prolonged follow-up period, are recommended to evaluate the long-term effectiveness of the outcomes. It is suggested that future comparative analysis of the Kinect-based VRT protocol and other treatment approaches in PMW with osteopenia be included, as well as a comparative study between PMW with osteopenia and those with osteoporosis. To account for potential confounding effects, it is recommended that future studies include demographic variables as covariates in their analysis. A follow-up study with the same treatment protocols in both groups but the control group playing unsupervised Kinect-based games at home will provide another dimension of this study. Evidence suggests that exercises utilizing Xbox 360 Kinect video games can enhance participants’ motivation and reduce fear avoidance. So, further research is needed to assess the impact on participants’ kinesiophobia and motivation, which may impact compliance with treatment. Moreover, future studies are suggested to incorporate wearable activity trackers to measure walking behavior in both groups objectively.

## 5. Conclusions

Both groups adhered to the SIOMMMS guidelines, participated in daily outdoor walking for 30 min, and demonstrated improved physical performance and quality of life. However, the experimental group, who received Kinect-based VRT in addition to walking, exhibited more substantial enhancements compared to the control group. The findings suggest that Kinect-based VRT provides additional benefits compared to walking alone in enhancing physical performance and quality of life in PMW with osteopenia.

## Figures and Tables

**Table 2:** Baseline Characteristics of Participants in both groups.

Variables	Experimental Group (N = 22)			Control Group (N = 21)			Intergroup Difference (*p*-Value)
	Minimum	Maximum	Mean ± SD	Minimum	Maximum	Mean ± SD	
Age of the Participants (years)	49.00	67.00	58.27 ± 5.13	49.00	68.00	58.00 ± 5.52	0.867
Height of the Participants (m)	1.44	1.72	1.64 ± 0.07	1.66	1.73	1.69 ± 0.022	0.060
Weight of the Participants (kg)	56.00	82.00	73.18 ± 7.0	67.00	88.00	78.48 ± 6.32	0.630
BMI of Participants (kg/m^2^)	21.30	32.90	27.42 ± 2.53	24.30	29.40	27.48 ± 1.45	0.921
Age At Menarche (years)	10.00	13.00	11.68 ± 0.89	10.00	13.00	11.47 ± 0.93	0.463
Age At Menopause (years)	48.00	54.00	49.77 ± 1.60	48.00	53.00	49.57 ± 1.66	0.688
Hip Circumference (cm)	102.00	117.00	110.68 ± 4.48	101.00	120.00	113.95 ± 4.08	0.316
Waist Circumference (cm)	80.00	95.00	89.41 ± 4.06	82.00	96.00	92.19 ± 3.23	0.305
Waist Hip Ratio	0.78	0.83	0.80 ± 0.014	0.78	0.83	0.81 ± 0.01	0.413
FES-I	20.00	28.00	23.32 ± 2.19	20.00	29.00	23.52 + 2.25	0.763
Serum Calcium	8.10	10.20	9.19 ± 0.57	8.30	9.90	9.02 + 0.42	0.282
Serum 25-OH Vitamin D	31.70	48.30	38.86 ± 6.04	25.20	48.70	38.86 + 5.43	0.999
Simple Calculated Osteoporosis Risk(SCORE)	6.00	15.00	8.55 ± 2.61	6.00	12.00	7.81 + 1.63	0.273

Abbreviations: SD = Standard Deviation, BMI = body mass index; FES-1 = Fall Efficacy Scale-1, SCORE = Simple Calculated Osteoporosis Risk Estimation.

**Table 3:** Frequency of demographic variables in both groups.

Variable	Categories	Experimental Group N (%)	Control Group N (%)
Marital Status	Married	21 (95.5)	19 (90.5)
	Unmarried	1 (4.5)	2 (9.5)
Low Back Pain (LBP)	Yes	22 (100)	21 (100%)
	No	0 (0)	0 (0%)
Bodily Pain	Yes	22 (100)	21 (100%)
	No	0 (0)	0 (0%)
Type II Diabetes	Yes	15 (68.2)	12 (57.1)
	No	7 (31.8)	9 (42.9)
Hypertension	Yes	15 (68.2)	13 (61.9)
	No	7 (31.8)	8 (38.1)
Use of contraceptives	Yes	6 (27.3)	1 (4.8)
	No	16 (72.7)	20 (95.2)
No. of coffee cups daily	Don’t take coffee	12 (54.5)	13 (61.9)
	One cup	8 (36.4)	6 (28.6)
	Two cups	2 (9.1)	2 (9.5)
	>two cups	0 (0)	0 (0)
No of tea cups daily	Don’t take tea	2 (9.1)	5 (23.8)
	One cup	5 (22.7)	6 (28.6)
	Two cups	9 (40.9)	7 (33.3)
	>two cups	6 (27.3)	3 (14.3)
History of the previous fracture	Yes	0 (0)	3 (14.3)
	No	100 (100)	18 (85.7)
Vitamin D intake	Yes	14 (63.6)	12 (57.1)
	No	8 (36.4)	9 (42.9)
Calcium intake	Yes	19 (86.4)	19 (90.5)
	No	3 (13.6)	2 (9.5)

**Table 4:** Intra-group and Inter-group comparisons of outcome variables at baseline,12th week, and 24th week.

Outcome Measures	Assessments	Experimental Group	Control Group	Pair-Wise Assessments	Experimental Group	Control Group	Intergroup Pairwise MD	Intergroup *p*-Value
		Mean ± SD	Mean ± SD		MD (95% CI), *p*-Value	MD (95% CI), *p*-Value	MD (95% CI)	
TUG	Baseline	10.20 ± 0.86	10.02 ± 0.63	Baseline–12th week	1.66 (1.45, 1.87), <0.001	0.21 (0.08, 0.35), 0.002	0.94 (0.82, 1.06)	<0.001
	12 weeks	8.54 ± 0.82	9.81 ± 0.64	12th week–24thweek	1.07 (0.86, 1.28), <0.001	0.31 (0.15, 0.46), <0.001	0.69 (0.56, 0.81)	<0.001
	24 weeks	7.48 ± 0.89	9.50 ± 0.71	Baseline–24th week	2.72 (2.40, 3.06), <0.001	0.52 (0.25, 0.79), <0.001	1.62 (1.42, 1.83)	<0.001
FRT	Baseline	23.58 ± 1.34	23.39 ± 0.96	Baseline–12th week	1.95 (2.21, 1.69), <0.001	0.15 (0.04, 025),0.005	1.05 (0.91, 1.19)	<0.001
	12 weeks	25.52 ± 1.26	23.54 ± 0.91	12th week–24thweek	3.15 (3.54, 2.75), <0.001	0.23 (0.05, 0.50), 0.126	1.69 (1.45, 1.92)	<0.001
	24 weeks	28.67 ± 1.46	23.76 ± 0.95	Baseline–24th week	5.09 (4.63, 5.51), <0.001	0.37 (0.03, 0.71), 0.029	2.73 (2.46, 3.0)	<0.001
FTSST	Baseline	10.24 ± 1.15	9.95 ± 0.69	Baseline–12th week	1.56 (1.32, 1.80), <0.001	0.19 (0.10, 0.30), <0.001	0.88 (0.75, 1.0)	<0.001
	12 weeks	8.68 ± 0.94	9.75 ± 0.63	12th week–24thweek	1.27 (1.02, 1.52), <0.001	0.25 (0.13, 0.37), <0.001	0.76 (0.62, 0.90)	<0.001
	24 weeks	7.41 ± 0.71	9.50 ± 0.68	Baseline–24th week	2.83 (2.47, 3.20), <0.001	0.44 (0.24, 0.65), <0.001	1.64 (1.44, 1.84)	<0.001
MSRT	Baseline	24.36 ± 1.63	23.26 ± 1.15	Baseline–12th week	1.95 (1.61, 2.29), <0.001	0.17 (0.06, 0.27), 0.002	1.06 (0.88, 1.23)	<0.001
	12 weeks	26.31 ± 1.58	23.43 ± 1.09	12th week–24thweek	2.80 (2.39, 3.21), <0.001	0.21(0.09, 0.32), 0.002	1.50(1.30, 1.71)	<0.001
	24 weeks	29.11 ± 1.36	23.63 ± 1.09	Baseline–24th week	4.75 (4.21, 5.29), <0.001	0.37 (0.16, 0.58), <0.001	2.56 (2.28, 2.84)	<0.001
DHGS	Baseline	22.76 ±1.59	22.82 ± 1.22	Baseline–12th week	2.0 (1.73, 2.29), <0.001	0.16 (0.05, 0.27), 0.044	1.08 (0.94, 1.23)	<0.001
	12 weeks	24.77 ± 1.66	22.98 ± 1.21	12th week–24th week	2.16 (1.81, 2.51), <0.001	0.31 (0.09, 0.52), 0.004	1.23 (1.03, 1.43)	<0.001
	24 weeks	26.92 ± 1.85	23.28 ± 1.34	Baseline–24th week	4.16 (3.65, 4.67), <0.001	0.47 (0.19, 0.75), 0.001	2.32 (2.03, 2.60)	<0.001
NDHGS	Baseline	20.69 ± 1.35	20.50 ± 1.24	Baseline–12th week	1.98 (1.67, 2.29), <0.001	0.13 (0.06, 0.20), 0.026	1.06 (0.90, 1.21)	<0.001
	12 weeks	22.67 ± 1.62	20.63 ± 1.20	12th week–24thweek	1.88 (1.44, 2.32), <0.001	0.22(0.08, 0.36),0.001	1.05(0.82, 1.28)	<0.001
	24 weeks	24.55 ± 2.04	20.85 ± 1.21	Baseline–24th week	3.86 (3.30, 4.42), <0.001	0.35 (0.16, 0.55), <0.001	2.11 (1.82, 2.40)	<0.001
BORG Score	Baseline	4.00 ± 0.69	2.95 ± 0.67	Baseline–12th week	1.14 (0.94, 1.33), <0.001	1.0 (1.0, 1.0), <0.001	0.88 (0.71, 1.04)	<0.001
	12 weeks	2.86 ± 0.77	1.95 ± 0.68	12th week–24th week	1.09 (0.85, 1.33), <0.001	0.27 (0.22, 0.55), 0.001	0.78 (0.61, 0.96)	<0.001
	24 weeks	1.77 ± 0.69	1.67 ± 0.73	Baseline–24th week	2.23 (1.99, 2.47), <0.001	1.29 (1.55, 1.02), <0.001	1.66 (1.50, 1.83)	<0.001
Dyspnea	Baseline	2.59 ± 0.50	2.43 ± 0.51	Baseline–12th week	1.0 (1.0, 1.0), <0.001	0.33 (0.06, 0.61), 0.015	0.67 (0.54, 0.80)	<0.001
	12 weeks	1.59 ± 0.50	2.09 ± 0.62	12th week–24th week	0.32 (0.58, 0.54), <0.001	0.67 (0.39, 0.94), <0.001	0.60 (0.41, 0.79)	<0.001
	24 weeks	1.27 ± 0.46	1.42 ± 0.51	Baseline–24th week	1.32 (1.05, 1.58), <0.001	1.0 (0.75, 1.26), <0.001	1.27 (1.07, 1.47)	<0.001
ECOS-16	Baseline	37.23 ± 1.66	36.62 ± 2.01	Baseline–12th week	4.96 (4.55, 5.35), <0.001	1.05 (0.71, 1.38), <0.001	3.0 (2.75, 3.25)	<0.001
	12 weeks	32.27 ± 1.55	35.57 ± 2.11	12th week–24th week	5.46 (4.76, 6.16), <0.001	1.68 (1.29, 2.04), <0.001	3.56 (3.18, 3.95)	<0.001
	24 weeks	26.82 ± 2.04	33.90 ± 2.32	Baseline–24th week	10.41 (9.58, 11.24), <0.001	2.71 (2.23, 3.19), <0.001	6.56 (6.10, 7.03)	<0.001

Abbreviations: MD = Mean Difference, CI = Confidence Interval, *p* = *p*-value (significant at <0.05), TUG = Time Up and Go, FRT = Functional reach Test, FTSST = Five Times Sit to Stand Test, MSRT = Modified Sit and Reach Test, DHGS = Dominant Hand Grip Strength, NDHGS = Non-Dominant Hand Grip Strength, ECOS-16 = Escala de Calidad de Vida Osteoporosis.

**Table 5:** Main effects (Group, Time) and interaction effects (Group*Time) for outcome variables.

Dependent Variable	Source	Mean Square	F	Partial Ƞ^2^	*p* Value
TUG	Group	34.34	58.30	0.32	<0.001
	Time	28.51	48.42	0.44	<0.001
	Group*Time	13.47	22.87	0.27	<0.001
FRT	Group	179.68	131.01	0.52	<0.001
	Time	81.71	59.58	0.49	<0.001
	Group*Time	60.93	44.43	0.42	<0.001
FTSST	Group	29.64	43.75	0.26	<0.001
	Time	28.84	42.56	0.41	<0.001
	Group*Time	15.45	22.81	0.27	<0.001
MSRT	Group	319.94	178.40	0.59	<0.001
	Time	71.11	39.65	0.39	<0.001
	Group*Time	52.08	29.04	0.32	<0.001
DHGS	Group	103.26	45.71	0.27	<0.001
	Time	57.67	25.53	0.29	<0.001
	Group*Time	36.69	16.24	0.21	<0.001
NDHGS	Group	123.39	54.67	0.31	<0.001
	Time	46.21	20.47	0.25	<0.001
	Group*Time	31.59	14.11	0.19	<0.001
BORG SCORE	Group	15.27	30.78	0.20	<0.001
	Time	33.67	67.85	0.53	<0.001
	Group*Time	2.78	5.61	0.08	0.005
DYSPNEA	Group	0.89	3.29	0.03	0.072
	Time	14.54	54.05	0.47	<0.001
	Group*Time	1.19	4.44	0.07	0.014
ECOS-16	Group	342.35	88.95	0.42	<0.001
	Time	463.72	120.48	0.66	<0.001
	Group*Time	159.05	41.33	0.40	<0.001

Abbreviations: TUG = Time Up and Go, FRT = Functional Reach Test, FTSST = Five Times Sit to Stand Test, MSRT = Modified Sit and Reach Test, DHGS = Dominant Hand Grip Strength, NDHGS = Non-Dominant Hand Grip Strength, ECOS-16 = Escala de Calidad de Vida Osteoporosis.

## Data Availability

The data generated or analyzed throughout this study is outlined in this paper and can be made available by the corresponding author upon a reasonable request.
